# Interactions between Zooplankton and Crude Oil: Toxic Effects and Bioaccumulation of Polycyclic Aromatic Hydrocarbons

**DOI:** 10.1371/journal.pone.0067212

**Published:** 2013-06-28

**Authors:** Rodrigo Almeda, Zoe Wambaugh, Zucheng Wang, Cammie Hyatt, Zhanfei Liu, Edward J. Buskey

**Affiliations:** 1 University of Texas Marine Science Institute, Port Aransas, Texas, United States of America; 2 Humboldt State University, Arcata, California, United States of America; 3 East China Normal University, Shanghai, China; University of Kansas, United States of America

## Abstract

We conducted ship-, shore- and laboratory-based crude oil exposure experiments to investigate (1) the effects of crude oil (Louisiana light sweet oil) on survival and bioaccumulation of polycyclic aromatic hydrocarbons (PAHs) in mesozooplankton communities, (2) the lethal effects of dispersant (Corexit 9500A) and dispersant-treated oil on mesozooplankton, (3) the influence of UVB radiation/sunlight exposure on the toxicity of dispersed crude oil to mesozooplankton, and (4) the role of marine protozoans on the sublethal effects of crude oil and in the bioaccumulation of PAHs in the copepod *Acartia tonsa*. Mortality of mesozooplankton increased with increasing oil concentration following a sigmoid model with a median lethal concentration of 32.4 µl L^−1^ in 16 h. At the ratio of dispersant to oil commonly used in the treatment of oil spills (i.e. 1∶20), dispersant (0.25 µl L^−1^) and dispersant- treated oil were 2.3 and 3.4 times more toxic, respectively, than crude oil alone (5 µl L^−1^) to mesozooplankton. UVB radiation increased the lethal effects of dispersed crude oil in mesozooplankton communities by 35%. We observed selective bioaccumulation of five PAHs, fluoranthene, phenanthrene, pyrene, chrysene and benzo[*b*]fluoranthene in both mesozooplankton communities and in the copepod *A. tonsa*. The presence of the protozoan *Oxyrrhis marina* reduced sublethal effects of oil on *A. tonsa* and was related to lower accumulations of PAHs in tissues and fecal pellets, suggesting that protozoa may be important in mitigating the harmful effects of crude oil exposure in copepods and the transfer of PAHs to higher trophic levels. Overall, our results indicate that the negative impact of oil spills on mesozooplankton may be increased by the use of chemical dispersant and UV radiation, but attenuated by crude oil-microbial food webs interactions, and that both mesozooplankton and protozoans may play an important role in fate of PAHs in marine environments.

## Introduction

Zooplankton play a key role in marine food web dynamics, biogeochemical cycling and fish recruitment [1]–[3]. However, despite their importance in marine environments, our knowledge of the interactions between zooplankton and anthropogenic pollutants is very limited. There are three main types of interactions between zooplankton and pollutants. First, pollutants can have direct toxic effects on zooplankton, including lethal or sublethal effects [4]. Second, zooplankton are able to influence the physicochemical characteristics of the pollutants in the water column (e.g. by absorption, transformation and elimination) [4]–[6]. Finally, zooplankton may play an important role in the biomagnification of pollutants up food webs [4], [7]. Therefore, understanding the interactions between pollutants and zooplankton is crucial for our understanding of the fate of pollution in the pelagic zone and their impact on marine environments.

Petroleum or crude oil is one of the most common pollutants released into the marine environment. Natural petroleum seeps, extraction, transportation, and consumption are the main sources of crude oil to the sea [8]. Although oil spills represent a small fraction of the total crude oil discharge into the sea, they have strong acute and long-term impacts on marine ecosystems, including effects from physical damages (physical contamination and smothering) and toxicity of their chemical compounds [8]. Recently, the Deepwater Horizon (DWH) oil spill in the Gulf of Mexico has raised concerns about the dramatic environmental and socio-economic impacts caused by oil spills in marine and coastal environments [9]–[11]. Crude oil is a complex mixture of both hydrocarbons, such as alkanes, cycloalkanes and aromatic hydrocarbons, and non-hydrocarbon compounds. Polycyclic aromatic hydrocarbons (PAHs) are considered to be the most acutely toxic components of crude oil, exerting its toxicity by interfering with membrane fluidity [12]. PAHs are also associated with potential carcinogenic, teratogenic and mutagenic effects in aquatic animals and humans [13]–[16]. After an oil spill, small crude oil droplets (1–100 µm in diameter) generated by waves and winds are effectively suspended in the water column [17], [18]. Also, plumes of small stable dispersed oil droplets are frequently found in subsurface waters after oil spills are treated with dispersants [19]. These crude oil droplets, which are frequently in the food size spectra of many zooplankters, can easily interact with planktonic organisms. For instance, small crude oil droplets can be ingested by zooplankton (protozoan and metazoans) when they are suspended in the water or attached to phytoplankton [20]–[26].

Among zooplankton, mesozooplankton (200–2000 µm) occupy a key position in pelagic food webs because of their role in the transfer of matter from primary producers to higher trophic levels [27], [28]. Copepods are the dominant group of mesozooplankton in marine environments [27]. Lethal and sublethal effects, including narcosis [29], alterations in feeding [30], development [31], and reproduction [32]–[34] have been observed in copepods exposed to petroleum hydrocarbons. Effects of petroleum hydrocarbons on mesozooplankton (e.g. copepods) vary widely depending on intrinsic (e.g., species, life stage, size) and extrinsic factors (e.g., oil concentration, exposure time, temperature) [30], [35]–[38]. Field and laboratory studies have also shown that copepods can accumulate PAHs [22], [25], [39]–[41]. Most crude oil toxicity tests and PAH bioaccumulation studies on zooplankton have been conducted using the crude oil water soluble fraction (WSF), or certain mixed or individual PAHs. However, since zooplanktons can ingest oil droplets [20], [24], [25], exposure to dispersed crude oil may promote the uptake of PAHs as compared with experiments using WSF. For example, the concentration of PAHs in fish was higher in fish exposed to dispersed crude oil than when exposed to WSF at the same hydrocarbon concentration [42]. Moreover, toxicity test and PAH bioaccumulation studies have traditionally focused on single species and conducted in the absence of food (starvation) [29], [43]. Therefore, experiments with natural mesozooplankton assemblages exposed to suspended crude oil with natural food conditions are required to better estimate the potential accumulation of petroleum hydrocarbons by zooplankton and their toxic effects.

Treatment of oil spills frequently involves the use of dispersants, which are mixtures of surfactants and other soluble compounds. Dispersants promote the removal of an oil slick from the surface waters enhancing the formation of small oil droplets, and therefore increasing their rate of natural dispersion. The first types of dispersants, like those used in the Torrey Canyon (1967) and Sea Empress (1996) oil spills, were highly toxic to marine animals, including fish, bivalves, and crustaceans, according to laboratory studies and field observations [44]–[48]. New types of dispersants (e.g. Corexit series dispersants, Corexit 9500 and Corexit 9527) are less toxic than the older types and have low to moderate toxicity to most marine animals according to laboratory studies [49], [50]. Thus, it has been suggested that the new generation of dispersants and dispersant treated - oil are less toxic than the spilled oil alone [51], [52] and that they have minimal deleterious effects on marine life [53]. However, little is known about the effects of this dispersant or dispersant treated oil on copepods or natural mesozooplankton communities, even though they are particularly susceptible to oil/dispersant exposure and they have important roles in marine ecosystems.

Most oil toxicological studies during the last decades have been conducted in the laboratory under artificial, fluorescent light [54]. However, there is increasing evidence that sunlight, mainly UV radiation (UVR), can increase the toxicity of petroleum hydrocarbons to marine organisms [55]–[58]. Photoenhanced toxicity (i.e., increase in the toxicity in the presence of light) of certain petroleum hydrocarbons has been observed in certain marine organisms [55]–[57], but information on phototoxicity of crude oil in zooplankton is scarce [59]. Therefore, knowledge of the effects of combined UVR and oil/dispersed oil/dispersant on zooplankton communities is essential for a better understanding of the impact of oil spills in the ocean.

Protozoan microplankton (e.g. ciliates and heterotrophic dinoflagellates) are the major consumers of phytoplankton and are important contributors to the diet of copepods [60]. Protozoans can also ingest oil droplets [21] and oil-contaminated phytoplankton. Bioaccumulation of PAHs in copepods may increase by feeding on oil-contaminated protozoans, but protozoans may also remove oil from the water, reducing the oil available for copepods. Therefore, in natural planktonic communities, the influence of crude oil on copepods may be affected by complex interactions between crude oil and microbial communities, including protozoans. Nevertheless, the potential role of protozoans in the interactions between dispersed crude oil and copepods (e.g. biomagnification or mitigation) has generally been neglected in petroleum toxicological and bioaccumulation studies.

The overall goal of this study was to improve our knowledge of the interactions between crude oil and marine zooplankton. To address this topic we conducted 3 types of experiments: 1) ship-based crude oil exposure experiments with natural mesozooplankton assemblages from the northern Gulf of Mexico, 2) shore-based crude oil and dispersant-treated crude oil exposure experiments with coastal mesozooplankton communities, and 3) laboratory crude oil exposure experiments with the copepod *Acartia tonsa*. *A. tonsa* is a widespread and dominant calanoid copepod species in estuaries and coastal waters, including the Gulf of Mexico. The specific objectives were to (1) determine the effects of short-term crude oil exposure on the survival and bioaccumulation of PAHs in natural mesozooplankton assemblages; (2) assess the lethal effects of dispersant-threated crude oil and dispersant (Corexit EC9500A) on coastal mesozooplankton communities; (3) estimate the influence of UVB radiation/sunlight exposure on the toxicity of dispersed crude oil to mesozooplankton communities; and (4) examine the role of marine protozoans on the sublethal effects (i.e., egg production, egg hatching, and egestion rates) of crude oil and the bioaccumulation of PAHs in the copepod *A. tonsa.* We used *Oxyrrhis marina,* a cosmopolitan heterotrophic dinoflagellate common in many intertidal and coastal habitats, as a model marine protozoan.

## Methodology

### Experimental Organisms

**Figure 1:**
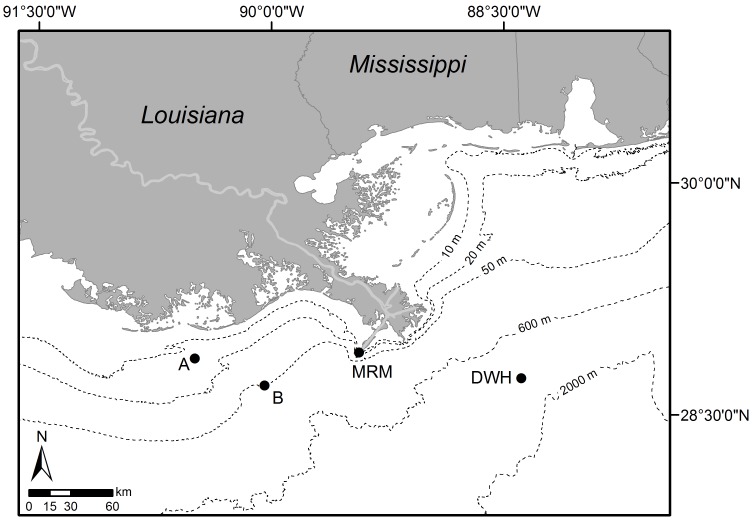
Map indicating the zooplankton sampling stations during the cruise in the northern Gulf of Mexico: station A (A), station B (B) and Mississippi River Mouth station (MRM). Stations are located in the area affected by the deepwater horizon (DWH) oil spill on April 2010.

Natural zooplankton assemblages were collected from 3 stations in the northern Gulf of Mexico on the research vessel “Pelican” in May 2012 during a four-day cruise (Fig. 1) and from the Aransas Ship Channel near the University of Texas Marine Science Institute (MSI) in Port Aransas, TX (27°49′39″ N 97°4′20″W). No permission is required for collecting zooplankton within state (Texas) or federal waters in our sampling areas. The University of Texas does not require an Animal Use/Animal Care protocol for invertebrates (only for vertebrates). Our studies did not involve endangered or protected species.

During the cruise, zooplankton samples were obtained by slow-speed plankton tows (10 m min^−1^) using a plankton net (50 cm diameter, 150 µm-mesh) with a 3 L plastic bag as a non-filtering cod end in order to minimize capture stress and physical damage to the organisms. Vertical tows from near the bottom to the surface were conducted at stations A (18 m depth) and B (50 m depth). In the station MRM (Mississippi River Mouth, 6 m depth), zooplankton samples were collected by horizontal tow from surface water. Zooplankton samples from the Aransas Ship Channel were collected from surface waters by tying the plankton net to the MSI pier and allowing it to stream with the tidal current for approximately 5–10 min. The plastic bags were kept in isothermal containers with seawater at in situ temperature until returning to the laboratory. Natural zooplankton assemblages were gently screened through a 2000 µm mesh sieve to remove large zooplankton (e.g. chaetognaths, salps, scyphozoans). Then, the mesozooplankton sample was carefully concentrated with a 150 µm mesh sieve and placed into a glass beaker with 0.2 µm-filtered seawater.

The calanoid copepod *Acartia tonsa* was collected in Aransas Bay (Texas coast) using a similar plankton net as used for the natural zooplankton assemblages. In the laboratory, approximately 100 adults (males and females) were sorted under a stereomicroscope and placed into a beaker with filtered sea water (FSW). To reduce the presence of other planktonic organisms, adult *A. tonsa* were repeatedly transferred through a series of petri dishes with 0.2 µm FSW. Specimens were reared in the laboratory for several weeks in 25 L transparent glass tanks with 1 µm FSW at 25°C under a 12-hour day/night cycle. *A. tonsa* cultures were fed the cryptophyte *Rhodomonas* sp. (equivalent spherical diameter, ESD = 7 µm), which were grown at 24°C in 10 L glass flasks using ‘f/2′ medium. The heterotrophic dinoflagellate *Oxyrrhis marina* (ESD = 15 µm), was fed with *Rhodomonas* sp. and cultured in 2 L glass beakers at similar temperature and light regime.

### Preparation of Crude Oil Emulsions and Dispersant Treated-oil

In this study, we used a Light Louisiana Sweet Crude Oil and determined the concentration and composition of PAHs in this oil. This crude oil was provided by BP (BP Exploration & Production Inc.) as a surrogate for the Macondo (MC252) crude oil released in the Deepwater Horizon oil spill in the Gulf of Mexico because they are considered to have similar chemical composition and toxicity. Corexit 9500A, one of the Corexit series of oil spill dispersant, was used for the shore-based experiments. The dispersant was provided by NALCO (Nalco/Exxon Energy Chemicals, L.P.) and its chemical composition can be found in the NALCO web page [61].

To prepare crude oil-seawater emulsions (i.e. suspensions of oil droplets in seawater), 0.2 µm filtered seawater was placed in a glass beaker with a magnetic stir bar, which was tightly sealed with aluminum foil to prevent oil absorption on the surface of the bar. Crude oil was added to the seawater using a Hamilton steel plunger microliter syringe and the glass beaker was placed on a magnetic stirrer plate. After covering the beaker with Teflon film, the oil was emulsified by stirring at 900 rpm for 5 min at room temperature (25°C). This stir speed allowed the formation of a vortex large enough to generate oil droplets in seawater. The formation of oil droplets, most of them between 1–10 µm of diameter, was confirmed using an Imaging Particle Analysis system (FlowCAM). To prepare dispersant threated-oil, we used a ratio of dispersant to oil of 1∶20, which is in the range (1∶50–1∶10) recommended by U.S. EPA [62].

### Experimental Design and Procedures

We conducted ship-based crude oil exposure experiments to investigate the effects of crude oil on survival and bioaccumulation of PAHs in mesozooplankton from the northern Gulf of Mexico. Natural mesozooplankton assemblages (community-based approach) were incubated onboard with natural seawater, which contained emulsified crude oil at a concentration between 10–100 µl L^−1^ (Table 1). Each experiment consisted of three replicates at each crude oil concentration (“experimental bottles”) and three control treatments (no crude oil added, “control bottles”). Water for these incubations was collected from Niskin bottles from the deep chlorophyll maximum (DCM, surface waters during this cruise) and transferred directly into acid-washed 1 L polycarbonate bottles with silicon tubing using a 3-step filling procedure to ensure homogeneity between replicates. Sea water samples (4 L) from the DCM were filtered through pre-incinerated GF/F filters and frozen (-20°C) for further analysis of polycyclic aromatic hydrocarbons as the background level. Aliquots from the zooplankton concentrate sample were added to the experimental and control bottles. Two additional aliquots were preserved in 4% buffered formaldehyde for later analysis of the initial copepod composition and concentration. After adding emulsified oil to the corresponding experimental bottles, bottles were incubated on deck in a large transparent acrylic container mounted to a plankton wheel with open-circuit seawater from 5-m depth running through it, thus providing exposure to sunlight and *in situ* temperature. The water temperature during the incubations was 25.5°C. After 16 hours of incubation, the contents of each bottle were gently screened through a submerged 150 µm mesh sieve to collect the zooplankton. Zooplankton were then rinsed 2 times with FSW, concentrated and placed in a beaker with 220 ml FSW. One aliquot with at least 20 individuals was placed in Petri dishes filled with 0.2 µm filtered seawater and then, checked for swimming activity and survival after 5 min. After 1 hour of being removed from the crude oil, we checked the copepods again for signs of recovery. One aliquot (20 ml) for the zooplankton concentrate was preserved in 4% buffered formaldehyde for later analysis of the final copepod species composition and abundance. The remaining sample was filtered again using a 150 µm mesh sieve and thoroughly rinsed with surface seawater using a pressure hose to minimize oil droplets that could potentially be attached to the copepods. Then, the rinsed copepod samples were filtered onto pre-combusted (450°C, 6 h) glass-fiber filters (GF/F) and frozen (−20°C) until further hydrocarbon analysis. For the estimation of abundance and species composition of natural mesozooplankton assemblages, one aliquot of at least 100 organisms from each sample was examined under a stereomicroscope.

**Table 1:** Initial mesozoplankton concentration (ind. L^−1^) and composition in the crude oil exposure experiments conducted in the northern Gulf of Mexico (Stations A, B and MRM) and in the Aransas Ship Channel (AC1 and AC2).

*Taxonomic groups/Category*	*Stations*				
	A	B	MRM	AC1	AC2
**Calanoid copepods**					
*Acartia tonsa*	395	7	901	104	73
*Paracalanus spp*	379	234	0	32	75
*Parvocalanus crassirostris*	1	0	50	29	8
*Calocalanus spp*	3	95	0	0	0
*Centropages spp*	74	3	2	0	0
*Euchaeta spp*	0	48	0	4	0
*Temora spp*	11	22	0	32	0
Others	44	69	0	18	5
**Cyclopoid copepods**					
*Oithona plumifera*	0	83	0	0	0
*Oithona spp*	30	5	442	36	48
**Poecilostomatoid copepods**					
*Oncaea spp*	27	379	12	0	0
*Corycaeus sp*	29	76	0	0	0
*Farranula sp*	0	12	0	0	0
**Harpacticoid copepods**					
*Euterpina acutifrons*	23	0	4	4	8
*Microsetella sp*	6	17	0	0	0
Others	0	0	0	0	3
**Copepod Nauplii**	7	52	10	11	23
**Other holoplankton**					
*Oikopleura dioica*	10	2	0	4	18
*Mysidacea larvae*	17	5	0	0	0
Others	11	8	0	0	0
**Meroplankton**					
*Polychaeta*	0	0	0	32	10
*Gastropoda*	0	10	0	0	38
*Cirripedia*	1	0	8	36	48
Other larvae	1	2	0	0	8
**Total (ind L** ^−**1**^ **)**	**1069**	**1129**	**1429**	**342**	**365**

We conducted two shore-based crude oil exposure experiments (community-based approach). In the first experiment, coastal mesozooplankton communities were incubated in quartz bottles (exposed to the full solar radiation spectrum) with crude oil (5 µl L^−1^), dispersant (0.25 µl L^−1^) and crude oil+dispersant (20∶1) for 48 h to determine the lethal effect of dispersant-treated oil and dispersant on mesozooplankton communities. Control and experimental treatments were performed in triplicates. In the second experiment, mesozooplankton communities were incubated in quartz bottles with dispersant treated oil (5 µl L^−1 ^oil +0.25 µl L^−1^ dispersant) for 48 h under 3 different light regimes: the full solar radiation spectrum (PAR+UVR), the full spectrum without UVB (i.e., PAR+UVA, covered with Mylar-D foil) and kept in the dark (covered with aluminum foil) to assess the effect of UVR/sunlight in dispersed oil toxicity. Control and experimental treatments were run in duplicates. In both experiments, mesozooplankton communities were incubated with natural seawater collected from surface waters. Experimental procedures used to determine mortality were similar to those described above for the ship-based experiments. Bottles were incubated on the MSI pier in a large open/uncovered transparent acrylic container containing a plankton wheel with open-circuit seawater running through it, thus providing exposure to sunlight and *in situ* temperature. Temperature and light were measured using a YSI® Model 30 SCT Meter and a LI-COR® LI-250A Light Meter, respectively. In the first experiment, water temperature was 19°C (±3°C) and the measured solar radiation ranged from 48 to 485 µmol photons m^−2^ s^−1^ during the daylight hours. In the second experiment, water temperature was 18°C (±1°C) and the measured solar radiation ranged from 122 to 757 µmol photons m^−2^ s^−1^ during the daylight hours. Survival of mesozooplankton in the different treatments was estimated as describe above for the ship-based experiments.

We conducted laboratory crude oil exposure experiments to evaluate the role of marine protozoans on the sublethal effects of crude oil and the bioaccumulation of PAHs in the copepod *Acartia tonsa.* Adult stages of *A. tonsa* were incubated with crude oil (5 µl L^−1^) in the laboratory for 48h. Two types of incubations experiments were conducted: 1) *A. tonsa* fed with a phytoplankton species, *Rhodomonas* sp. and 2) *A. tonsa* fed with *Rhodomonas* sp. and a protozoan species, *Oxyrrhis marina.* Each experiment included triplicate experimental treatments (“experimental“) and 1–2 control treatments (“control”). Adult *A. tonsa* were removed from stock cultures by filtering them through a submerged 150 µm mesh sieve and were concentrated in FSW. Aliquots containing approximately 600 adult copepods were then placed into glass aquariums containing 15 L of FSW and the 2 different food regimes, *Rhodomonas* sp. (50,000 cells mL^−1^) and *Rhodomonas* sp.+*Oxyrrhis marina* (50,000 cells mL^−1^+700 cells mL^−1^, respectively). Next, oil emulsions were added to the corresponding experimental aquariums. To keep the oil droplets suspended in the water, turbulence was created by aeration using 2 glass tubes connected to an air pump. Experimental and control (without oil) treatments were run in duplicate, simultaneously. Incubations were conducted at 25°C under artificial dim light for 48 h. After incubation, two aliquots with at least 25 individuals from each aquarium were placed in Petri dishes filled with 0.2 µm filtered seawater and then checked for swimming activity and survival. Next, all *A. tonsa* adults from each aquarium were separated out from water, which contains their fecal pellets and eggs, using a 150 µm mesh sieve. As with the community-based approach, the samples were thoroughly rinsed with FSW using a pressure sprayer and concentrated in 400 ml of FSW. To separate copepod eggs from fecal pellets, water samples (fraction <150 µm) were screened through a 40 µm mesh sieve, rinsed thoroughly using a pressure sprayer and concentrated in 200 mL of FSW. The separation of eggs from fecal pellets was corroborated under a stereomicroscope. Finally, fecal pellets/debris were filtered using a 20 µm mesh sieve, rinsed and concentrated in 400 mL of FSW. One aliquot (10 or 15 ml) of each type of concentrated sample (copepod, eggs or fecal pellets) was preserved in 1% Lugol’s solution for counting. The remaining concentrated samples of the copepod, eggs and fecal pellets were filtered onto pre-combusted (450°C, 6 h) glass-fiber filters (GF/F) and frozen (−20°C) until further hydrocarbon analysis.

### Chemical Analysis

Sixteen priority PAHs defined by the US Environmental Protection Agency (EPA) were analyzed: naphthalene (Nap), acenaphthene (Ace), acenaphthylene (Acy), fluorene (Flu), phenanthrene (Phe), anthracene (An), fluoranthene (Flua), pyrene (Pyr), benzo[*a*]anthracene (BaA), chrysene (Chr), benzo[*b*]fluoranthene (BbF), benzo[*k,*j]fluoranthene (BkF), benzo[*a*]pyrene (BaP), indeno [*1]*, [2], *[3*]pyrene (InP), dibenzo[*a,h*]anthracene (DBA), and benzo[*ghi*]perylene (BgP). The 16 PAH standards and 3 PAH surrogate standards (D_10_- Acenaphthene (Ace-D_10_), D_10_Phenanthrene (Phe-D_10_), D_12_-Benzo[a] anthracene (BaA-D_12_) were purchased from Sigma. All organic solvents (HPLC grade) were purchased from Fisher Scientific. Sodium sulfate and neutral alumina were baked at 450°C for 4 h. The silica gel was cleaned with dichloromethane (DCM) before using. The neutral alumina and silica gel were activated by heating at 120°C for 12 h. Reagent grade water (5% wt.) was mixed with the neutral alumina for partial deactivation.

**Figure 2:**
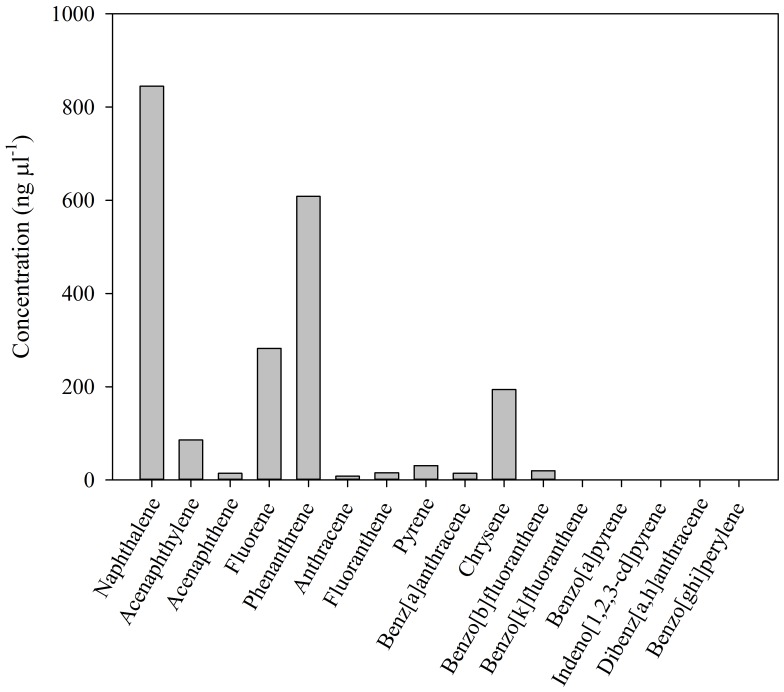
Concentration of polycyclic aromatic hydrocarbons (PAHs, ng µL^−1^) in the crude oil used in the experiments (Louisiana light sweet crude oil).

Chemical analysis of the crude oil followed the protocol of Liu et al. [63]. Briefly, 100 µL of crude oil was diluted to 1 mL with hexane. The sample was purified with a self-packed chromatographic column with 1g anhydrous sodium sulfate and 8 g silica gel. The column was eluted with 50 mL dichloromethane/hexane (1∶4, v/v). The eluted solution was concentrated to 1mL by a rotary evaporator, and preserved in a freezer (−20°C) until analysis by gas chromatography-mass spectrometry (GC/MS). The composition and concentration of PAHs in the Light Louisiana Sweet Crude Oil used in these experiments are shown in Figure 2.

Zooplankton samples were freeze-dried and weighed. Replicate samples were combined to obtain enough biomass for analysis. PAHs in zooplankton samples were extracted by Soxhlet extractors for 24 h, using hexane and DCM (1∶1, v/v) as the extraction solution. The solution was concentrated to ca. 2 mL by a rotary evaporator and purified with a chromatographic column packed with 1 g anhydrous sodium sulfate (top), 4 g neutral alumina (middle), and 8 g silica (bottom). The concentrated solution was eluted from the column with 50 mL DCM/hexane (1∶4, v/v). The collected solution was concentrated to 0.5 mL and exchanged with hexane by a rotary evaporator. A portion of the solution was used for the PAH analysis. PAHs were analyzed using GC/MS (Shimadzu QP2010 plus) with a RXi-1MS capillary column (20 m×0.18 mm i.d., film thickness 0.18 µm). The injection volume was 1 µL sample with a split ratio of 1/20, and the helium flow was set at 0.8 mL min^−1^. The temperatures of the injector and detector were set at 260°C and 275°C, respectively. The temperature of the column was ramped from 60°C to 240°C at 10°C min^−1^, and increased to 280°C at 4°C min^−1^ and held for 3 min. Selected ion monitoring mode was used to quantify PAHs, which ranged from 126 to 279 a.m.u., and dwell time per ion was 200 ms. The average recovery of surrogate standards for seawater and zooplankton were 93% (n = 12) and 95% (n = 12), respectively. The detection limit of this method is 0.001–0.004 ng/µL.

### Calculations

Mortality, as % of the incubated organisms, was estimated from the number of dead (not swimming after gently touching with a Pasteur pipette tip) individuals at the second visual checking. Narcosis (%) was estimated from the difference in the number of non-swimming individuals at the first checking (which included actual dead and narcotized animals) and the second checking (which included only those copepods that did not recover from toxic effects).

Data on copepod mortality versus crude oil concentration were fitted to the following sigmoid model:
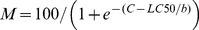
(1)where, M is the copepod mortality (%), *C* is the crude oil concentration (µl L^−1^), *LC_50_* is the median lethal concentration and *b* is the slope factor.

Egg production rates, fecal pellet production rates and egg hatching of *Acartia tonsa* were evaluated after 48 hours of crude oil exposure. Samples of adult stages, eggs/nauplii and faecal pellets of *A. tonsa* were counted under a stereomicroscope. Egg production was estimated as the total number of eggs and hatched eggs (nauplii). Hatching (%) was assessed from number of nauplii in relation to total number of observed eggs and nauplii after incubation time.

Bioaccumulation factor is the ratio of pollutant concentration in an aquatic organism to the water concentration that includes dietary uptake. The bioaccumulation factor (BAF) in the copepods exposed to crude oil was calculated as follows:
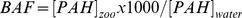
(2)where, *[PAH]_zoo_* is the concentration of polycyclic aromatic hydrocarbons (PAHs) in exposed copepods after subtracting the concentration of PAHs in the corresponding control treatment, in ng g^−1^ and *[PAH]_water_* is the concentration of PAHs in seawater, in ng L^−1^. Biomass was calculated as dry weight (DW). The concentration of PAHs in the water (Table 2) was estimated from the oil added to the containers, using the concentration of PAHs determined in the crude oil (Fig. 2). In our experiments, PAHs in the seawater would have been presented in both dissolved and particulate (oil droplet) forms.

**Table 2:** Concentration of polycyclic aromatic hydrocarbons, PAHs (µg L^−1^), in the water at the different crude oil exposure levels (5–100 µl L^−1^) used in the experiments.

[crude oil]µl L^−1^	[crude oil]mg L^−1^	Nap	Ace	Acy	Flu	Phe	An	Flua	Pyr	BaA	Chr	BbF
*5*	*4.2*	4.22	0.43	0.07	1.41	3.04	0.04	0.08	0.15	0.07	0.97	0.10
*10*	*8.5*	8.45	0.85	0.14	2.82	6.08	0.08	0.15	0.31	0.14	1.94	0.20
*20*	*16.9*	16.89	1.71	0.28	5.65	12.17	0.16	0.31	0.62	0.28	3.88	0.39
*25*	*21.1*	21.12	2.14	0.35	7.06	15.21	0.20	0.38	0.77	0.35	4.85	0.49
*30*	*25.4*	25.34	2.56	0.42	8.47	18.25	0.24	0.46	0.93	0.42	5.82	0.59
*50*	*42.3*	42.23	4.27	0.70	14.11	30.42	0.40	0.76	1.54	0.70	9.70	0.98
*100*	*84.5*	84.46	8.54	1.40	28.23	60.83	0.80	1.53	3.08	1.40	19.39	1.96

Concentration of PAHs was estimated from the oil added to the containers using the concentration of PAHs determined in the crude oil (Fig. 2) Crude oil exposure levels are also expressed in mg L^−1^ using a crude oil density of 0.845g/ml. Naphthalene (Nap), acenaphthene (Ace), acenaphthylene (Acy), fluorene (Flu), phenanthrene (Phe), anthracene (An), fluoranthene (Flua), pyrene (Pyr), benzo[a]anthracene (BaA), chrysene (Chr), benzo[b]fluoranthene (BbF).

## Results

### Composition of Natural Mesozooplankton Assemblages used in the Experiments

**Figure 3:**
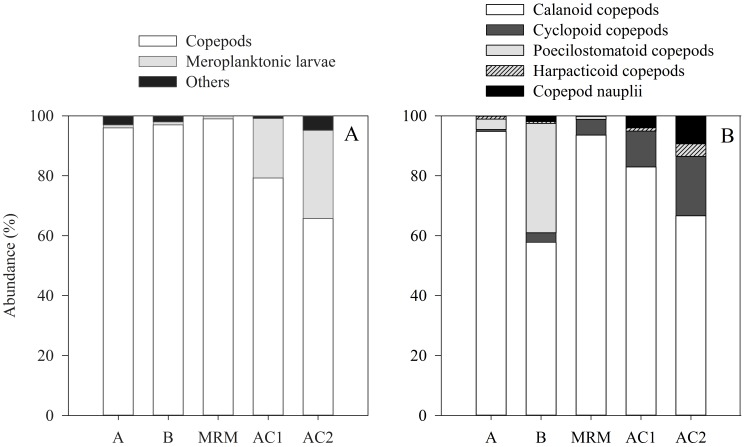
Composition in abundance (%) of the natural mesozooplankton assemblages used in the experiments. A: metazooplankton composition. B: copepod composition.

The natural mesozooplankton assemblages from northern Gulf of Mexico (Stations A, B and MRM) used in the experiments were dominated by copepods (96%–99%) (Fig. 3A). Calanoid copepods were the most abundant group of copepods at stations A and MRM, whereas both calanoid and poecilostomatoid copepods were the major components of the copepod community at station B (Fig. 3B). We observed differences in copepod taxonomic composition among stations in the northern Gulf of Mexico (Table 1). Stations C6 and NC had a high diversity of copepod species, whereas at station MRM the copepod community was mainly dominated by the calanoid copepod *Acartia tonsa* and the cyclopoid copepod *Oithona spp* (Table 1). Mesozooplankton communities from the Aransas Ship Channel (AC1, AC2) were also dominated by copepods but meroplanktonic larvae represented ca. 20–30% in abundance (Fig. 3A). The main meroplanktonic larvae were cirripede nauplii, polychaeta larvae, and gastropod veligers. Calanoids (e.g. *Acartia, Paracalanus, Parvocalanus, Temora*) and cyclopoids (*Oithona* spp.) were the main groups of copepods observed in the mesozooplankton communities from the Aransas Ship Channel (Table 1).

### Lethal Effects of Crude Oil on Northern Gulf of Mexico Mesozooplankton Communities

**Figure 4:**
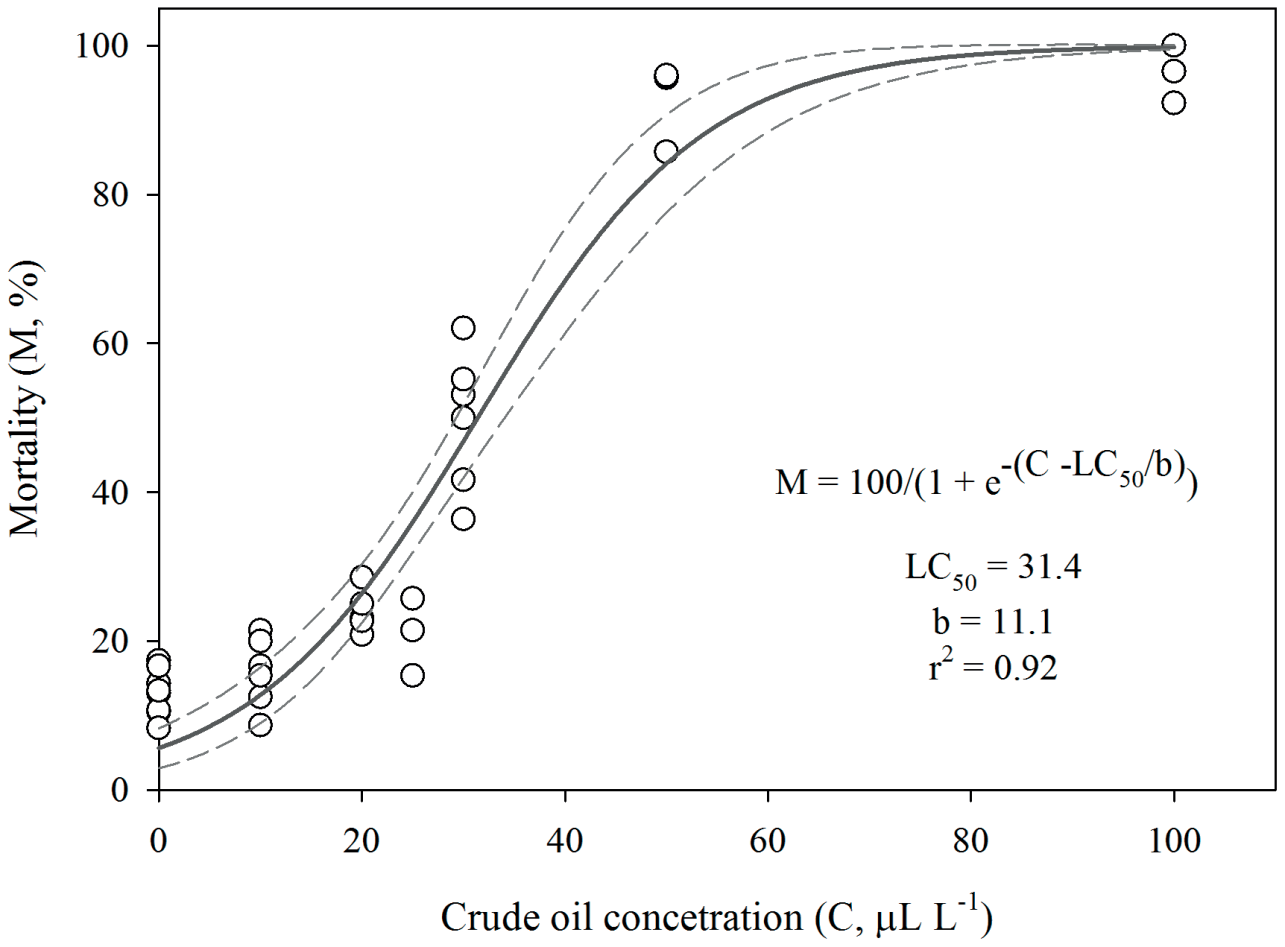
Relationship between mesozooplankton mortality and crude oil concentration after 16 h of exposure in onboard incubations (25°C, sunlight exposure) conducted in the northern Gulf of Mexico. Regression line based on Equation (1) (solid line) and 95% confidence intervals (dashed lines).

Overall, we observed a significant effect of crude oil on mesozooplankton survival (ANOVA, F_6, 29_ = 181.9, p<0.01; Table 3). Mortality ranged from 12% to 96% depending on crude oil concentrations and station (Table 3). At each station, average mesozooplankton mortality (%) increased as crude oil concentrations increased (Table 3). At station A, massive mesozooplankton mortality (>90%) was observed at crude oil concentrations ≥50 µl L^−1^ after only 16 h (Table 3). By including data from all experiments, the relationship between mesozooplankton mortality (%) and crude oil concentration was well described by the sigmoid model (r^2^ = 0.92) (Fig. 4). According to the model, the median lethal concentration (LC_50_), i.e. lethal concentration required to kill half the members of a tested population, was 31.4 µl L^−1^ after 16 h (Fig. 4). Narcosis effects varied from 1% to 56% depending on the station and crude oil concentration (Table 3). Significant narcotic effects in mesozooplankton communities were observed at station A at crude oil concentration of 10 µl L^−1^ and at station MRM at all crude oil concentrations, where narcosis was higher than 50% at concentrations of 10 and 20 µl L^−1^ (Table 3).

**Table 3:** Mortality and narcosis of natural mesozooplankton communities from the northern Gulf of Mexico (Stations A, B and MRM) after 16 h of crude oil exposure.

Station	[crude oil]µl L^−1^	Mortality(%, Avg. ± SE)	Narcosis(%, Avg. ± SE)	n ± SD
A	0	14±2	7±1	25±3
	25	21±3	14±2*	30±5
	50	92±3*	2±2	23±2
	100	96±2*	1±1	24±6
B	0	12±1	4±2	25±5
	10	16±3	7±2	27±4
	20	23±1*	7±4	27±4
	30	55±3*	5±3	28±3
MRM	0	13±2	10±2	30±6
	10	16±2	56±4*	25±1
	20	25±2*	55±4*	22±2
	30	44±6*	35±7*	25±4

The asterisks indicate a significant difference (P<0.05) from respective controls. Avg.: average, SE: standard error.

### Lethal Effects of Dispersant and Dispersant-treated Oil on Mesozooplanton Communities

**Figure 5:**
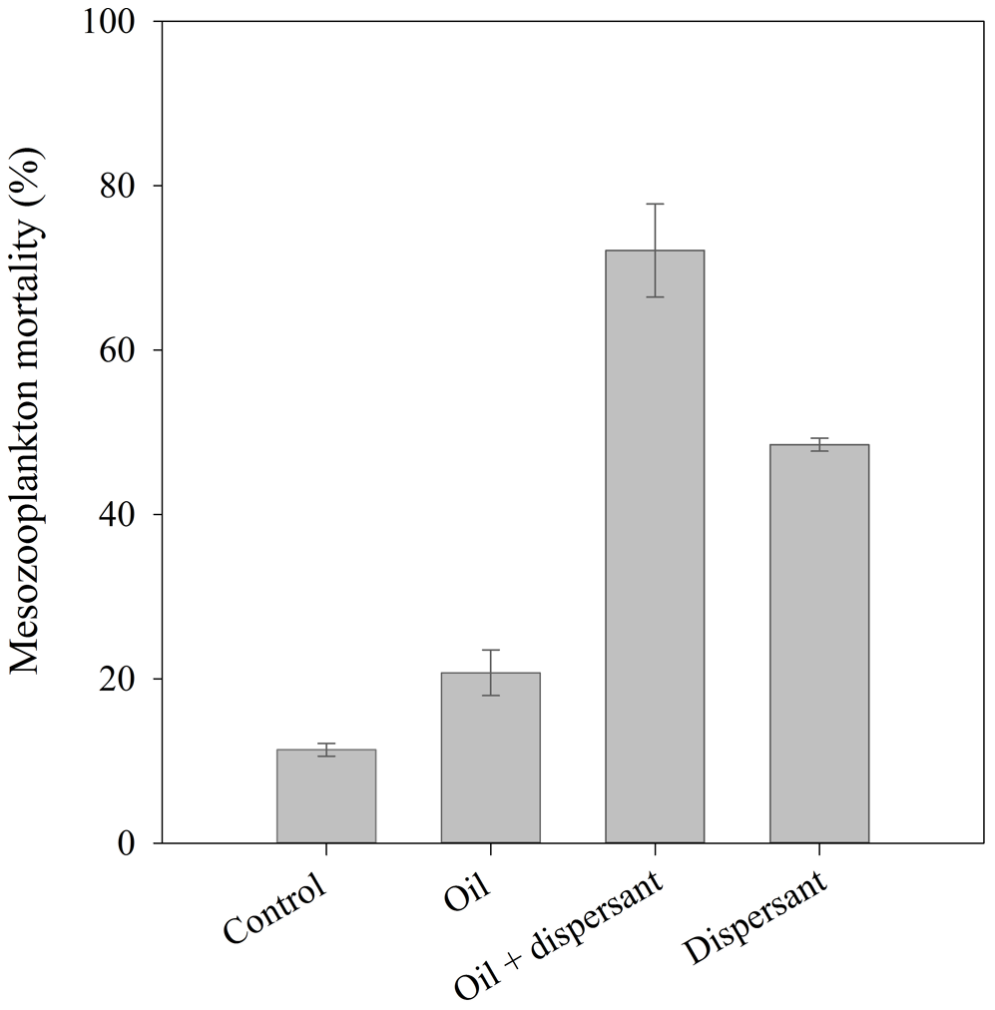
Lethal effects of crude oil (5 µl L^−1^), dispersant-treated crude oil, and dispersant (0.25 µl L^−1^) on mesozooplanton communities from the Aransas Ship Channel (AC1, Fig. 2) after 48 h incubation (T = 22°C, full solar radiation spectrum). Error bars represent the standard deviations.

We observed significant differences in mesozooplankton mortality among treatments (ANOVA, F = 149, p<0.01) (Fig. 5). Mortality in the control treatment was ca. 11%, significantly lower than in the experimental treatments (ANOVA, Tukey test, F = 149, p<0.01) (Fig. 5). Mortality of mesozooplankton communities exposed to crude oil (5 µl L^−1^) was 21% after 48 h (Fig. 5). Exposure of mesozooplankton communities to the dispersant (0.25 µl L^−1^) caused a mortality of 48% after 48h (Fig. 5). The highest mortality was observed in the dispersant-treated oil treatment, reaching values of 72% after 48 h (Fig. 5). Therefore, dispersant and dispersed-oil were >2.3 and >3.4 times more toxic, respectively, than crude oil alone to coastal mesozooplankton communities (Fig. 5).

### Influence of UV Radiation on the Toxicity of Dispersed Crude Oil to Mesozooplankton

**Figure 6:**
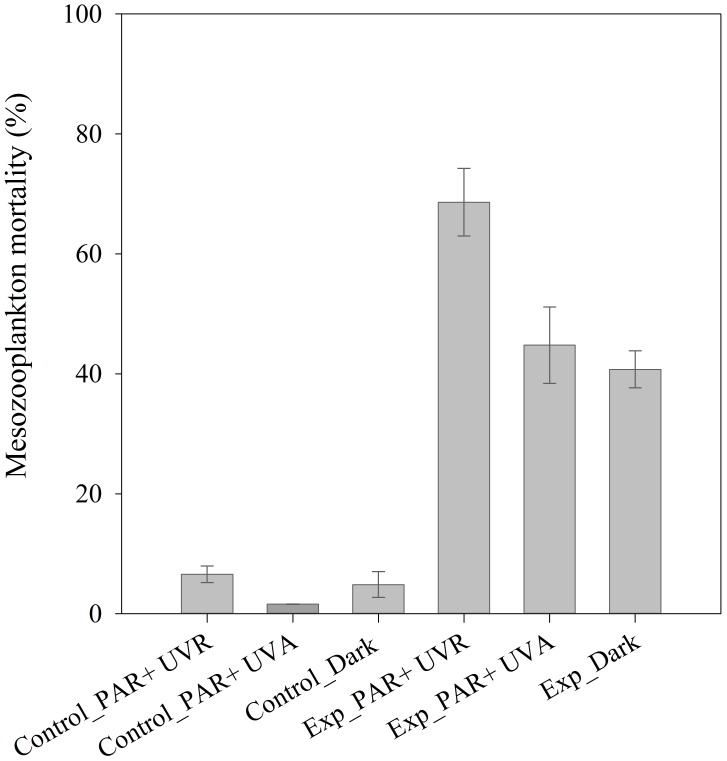
Lethal effects of dispersant-treated crude oil (5 µl L^−1^) on mesozooplanton communities from the Aransas Channel (AC2) under 3 different light regimes: the full solar radiation spectrum (PAR+UVR), the full spectrum without UVB (i.e., PAR+UVA) and kept in the dark after 48 h (T = 18°C). Error bars represent the standard deviations.

Mesozooplankton mortality was higher in experimental (5 µl L^−1^ of oil and 0.25 µl L^−1^ of dispersant) than in control treatments (no oil added) for the three different light regimes (ANOVA, p<0.01) (Fig. 6). Mortality was very low (<7%) in all control treatments (Fig. 6). Mesozooplankton mortality was lower in the control treatments without UVB radiation (‘Control_PAR+UVA’ and ‘Control _dark’) than in the control treatment exposed to the full solar radiation spectrum (‘Control_PAR+UVR’) (Fig. 6). Mortality of mesozooplankton exposed to dispersant-treated oil with the full solar radiation spectrum (‘Exp_PAR+UVR’) was 68.6% after 48 hours, significantly higher than with the other light regimes (‘Exp_PAR+UVA and ‘Exp_dark’) (ANOVA, F_2,3_ = 17.3, p<0.05) (Fig. 6). Mesozooplankton exposed to dispersant-treated oil without UVB radiation (‘Exp_PAR+UVA) and in the dark (‘Exp_dark’) showed a mortality of 44.8% and 40.7%, respectively, with no significant differences between treatments (ANOVA, F_1, 2_ = 0.5, p>0.05) (Fig. 6). These results indicated that UVA radiation had little influence in the toxicity of crude oil to mesozooplankton, and UVB radiation increased the lethal effects of dispersed crude oil to coastal mesozooplankton communities by 35% (Fig. 6).

### Sublethal Effects of Crude Oil Exposure on Acartia Tonsa

**Figure 7:**
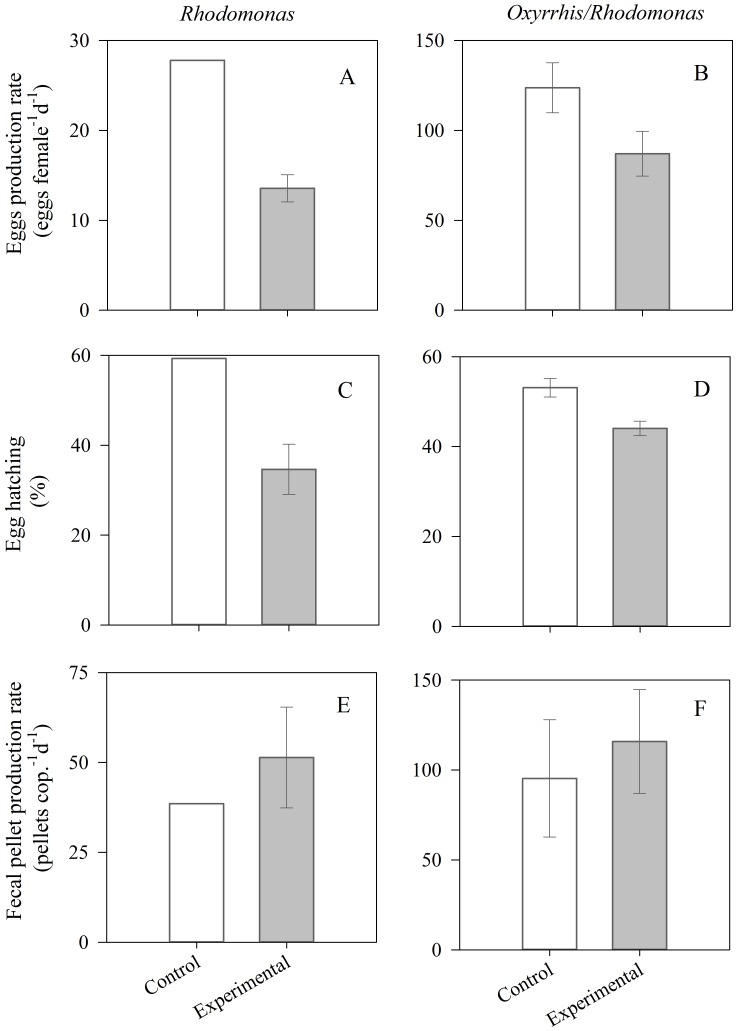
Effect of crude oil exposure (5 µl L^−1^, 48 h, dim light) on egg production rates, egg hatching and fecal pellet production rates of *Acartia tonsa* feeding on *Rhodomonas* sp. (left column, A, C, E) or *Rhodomonas* sp. plus *Oxyrrhis* marina (right column, B, D, F). Experimental: oil exposed copepods. Control: non-exposed copepods. Error bars represent the standard deviations.

In the laboratory experiments, mortality of *Acartia tonsa* was very low (0%–4%) after 48 hours of exposure (5 µl L^−1^), with no significant differences between experiment and control treatments (ANOVA, F_1, 8_ = 0.3, p>0.05). We did not observe narcotic effects in *Acartia tonsa* in these laboratory experiments. Egg production rates varied from 14–124 eggs female^−1^ d^−1^ depending on the food regime (*Rhodomonas* or *Oxyrrhis/Rhodomonas*) and the treatment (crude oil exposed or non-exposed copepods) (Fig. 7). Egg production rates were >4 times higher when *A. tonsa* was incubated with *Oxyrrhis*/*Rhodomonas* than when incubated only with *Rhodomonas* (Fig. 7A, 7B). In both food regimes, eggs production rates of *A. tonsa* exposed to crude oil were lower than in non-exposed individuals (Fig. 7A, 7B). The reduction in egg production rates was significantly lower (ANOVA, F_1, 5_ = 13.9, p<0.05) when *A. tonsa* was incubated with *Oxyrrhis/Rhodomonas* than when incubated only with *Rhodomonas* (1.42 and 2.05 times lower, respectively) (Fig. 7A, 7B). Egg hatching after 48 hours ranged from 39% to 59% depending on the food regime and treatment (Fig. 7C, 7D). As observed for egg production rates, egg hatching of *A. tonsa* exposed to crude oil was lower than control treatments for both food regimes (Fig. 7C, 7D). The reduction in egg hatching was significantly lower (ANOVA, F_1, 5 = _8.8, p<0.05) when *A. tonsa* was incubated with *Oxyrrhis*/*Rhodomonas* than when incubated with *Rhodomonas* (1.2 and 1.7 times lower, respectively) (Fig. 7C, 7D). Fecal pellets production rates ranged from 39–116 pellets ind^−1^ d^−1^ depending on the food regime and the crude oil treatment (Fig. 7E, 7F). Fecal pellet production rates were >2 times higher in *A. tonsa* incubated with *Oxyrrhis*/*Rhodomonas* than those incubated only with *Rhodomonas* (Fig. 7E, 7F). Fecal pellet productions rates of individuals not exposed to crude oil were lower than those exposed (Fig. 7E, 7F). However, fecal pellet productions rates showed high variability among replicates, and thus, non-significant differences (ANOVA, F_1,4_ = 0.6, p<0.05) between treatments were observed in *A. tonsa* incubated with *Oxyrrhis/Rhodomonas* (Fig. 7F).

### Bioaccumulation of PAHs in Natural Copepod Assemblages Exposed to Crude Oil

The total concentration of PAHs in the crude oil was 2.11 µg µL^−1^ (Fig. 2). Naphthalene, phenanthrene, fluorene, chrysene, and acenaphthylene were the most abundant PAHs in the crude oil (Fig. 2). The concentration of PAHs in the water used for the incubation experiments from all stations was undetectable in most cases, except for naphthalene.

**Figure 8:**
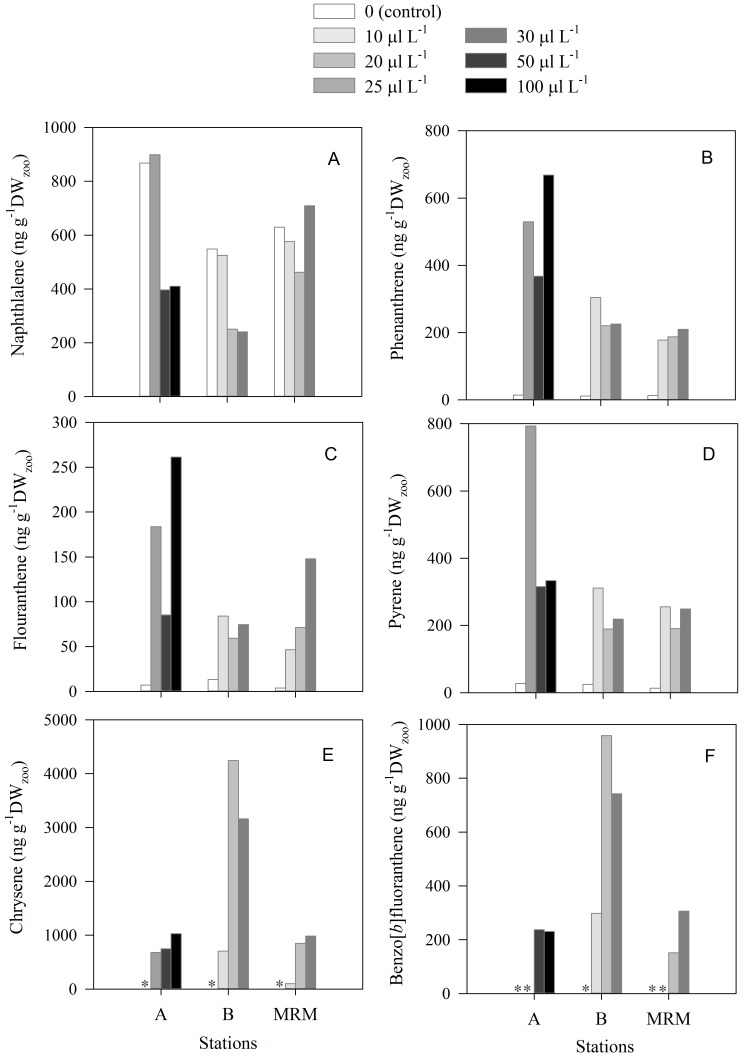
Concentration of the polycyclic aromatic hydrocarbons detected in natural copepod assemblages after 16 h of exposure to different crude oil concentrations (10–100 µl L^−1^) in the experiments conducted in the North of Gulf Mexico stations (A, B, MRM). A: naphthalene, B: phenanthrene, C: fluoranthene, D: pyrene, E: chrysene, F: benzo[b]fluorantheneThe asterisks indicate the PAH was not detected.

Naphthalene, fluoranthene, phenanthrene, pyrene, chrysene and benzo[*b*]fluoranthene were the main PAHs detected in copepods (Fig. 8). Total concentration of PAHs in copepods exposed to crude oil was between 2.5–10 times higher than those not exposed, depending on the station (Fig. 8). Except for naphthalene (Fig. 8A), the concentration of PAHs in copepods in the control treatment was very low at all stations, ranging from 0 for chrysene and benzo[*b*]fluoranthene (Fig. 8E, 8F) to <30 ng g^−1^ DW_zoo_ for fluoranthene, phenanthrene, pyrene (Fig. 8B, 8C, 8D). The concentrations of fluoranthene, phenanthrene, pyrene, chrysene were significantly higher (ANOVA, p<0.01) in copepods exposed to crude oil than in copepods not exposed to crude oil. At stations A and MRM, benzo[*b*]fluoranthene was not found in copepods at low crude oil concentration but was detected in copepods exposed to higher crude oil concentrations (Fig. 8F).

**Figure 9:**
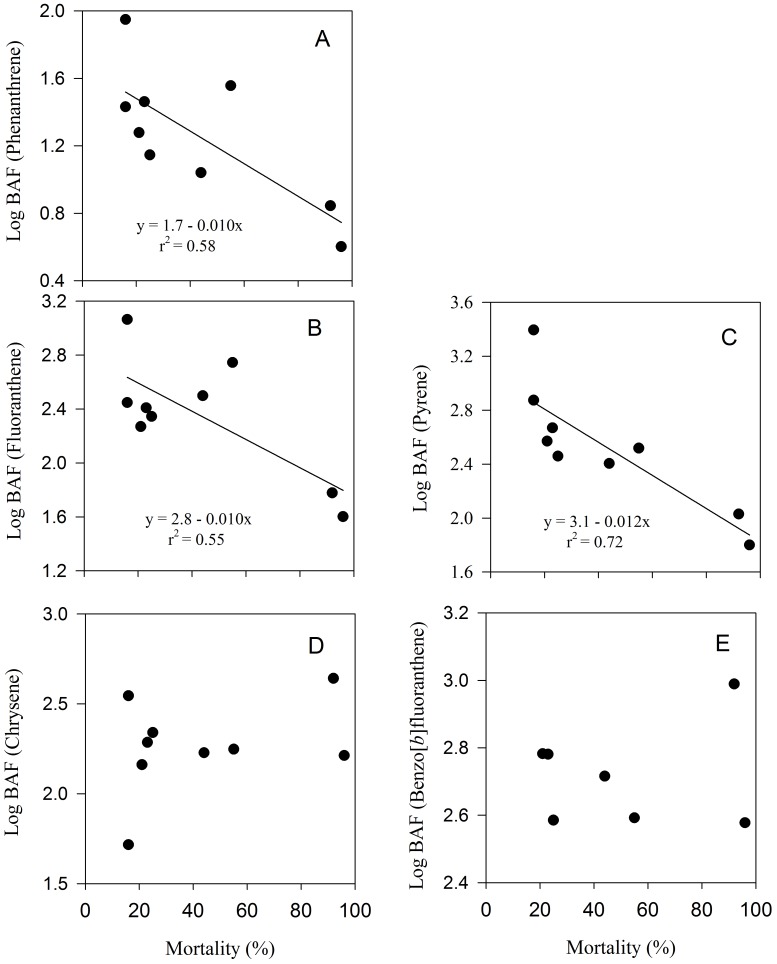
Relationship between bioaccumulation factors (BAF) and mortality (%) in natural copepod assemblages exposed to crude oil. A: phenanthrene, B: fluoranthene, C: pyrene, D: chrysene, E: benzo[b]fluoranthene.

Bioaccumulation factors (BAFs) ranged from 3 to 2570 depending on the type of PAH, the crude oil concentration and the copepod community (Table 4). BAFs for naphthalene and phenanthrene were lower than for the other PAHs (Table 4). The highest bioaccumulation factors (>1000) were for fluoranthene and pyrene in the copepods community from station B at crude oil concentrations of 10 µl L^−1^ (Table 4). At each station, we observed a decrease in BAFs for fluoranthene, phenanthrene, pyrene as crude oil concentrations increased (Table 4). Similarly, the BAFs for these PAHs decreased significantly as copepod mortality increased (Fig. 9 A–C). In contrast, we did not find any clear relationship between BAF of chrysene and Benzo[*b*]fluoranthene and crude oil concentration (Table 4) or copepod mortality (Fig. 9D, 9E).

**Table 4:** Bioaccumulation factors of PAHs in natural mesozooplankton communities from the northern Gulf of Mexico (Stations A, B and MRM) exposed to different concentrations of crude oil.

Stations	Oil conc.(µl L^−1^)	Nap	Phe	Flua	Pyr	Chr	BbF
A	25	4	19	186	372	145	606
	50	–	7	60	107	438	976
	100	–	4	40	63	163	378
B	10	–	89	1158	2482	351	n.d.
	20	–	29	256	467	193	604
	30	–	36	555	330	177	391
MRM	10	–	27	280	748	52	n.d.
	20	–	14	221	288	219	385
	30	3	11	315	254	169	520

Naphthalene (Nap), phenanthrene (Phe), fluoranthene (Flua), pyrene (Pyr), chrysene (Chr), benzo[b]fluoranthene (BbF). The hash symbol indicates that BAF were similar or lower than respective control treatments (non-exposed copepods). *n.d.* = no detected.

### Bioaccumulation of PAH in Tissues, Eggs and Fecal Pellets of A. Tonsa Exposed to Crude Oil

**Figure 10:**
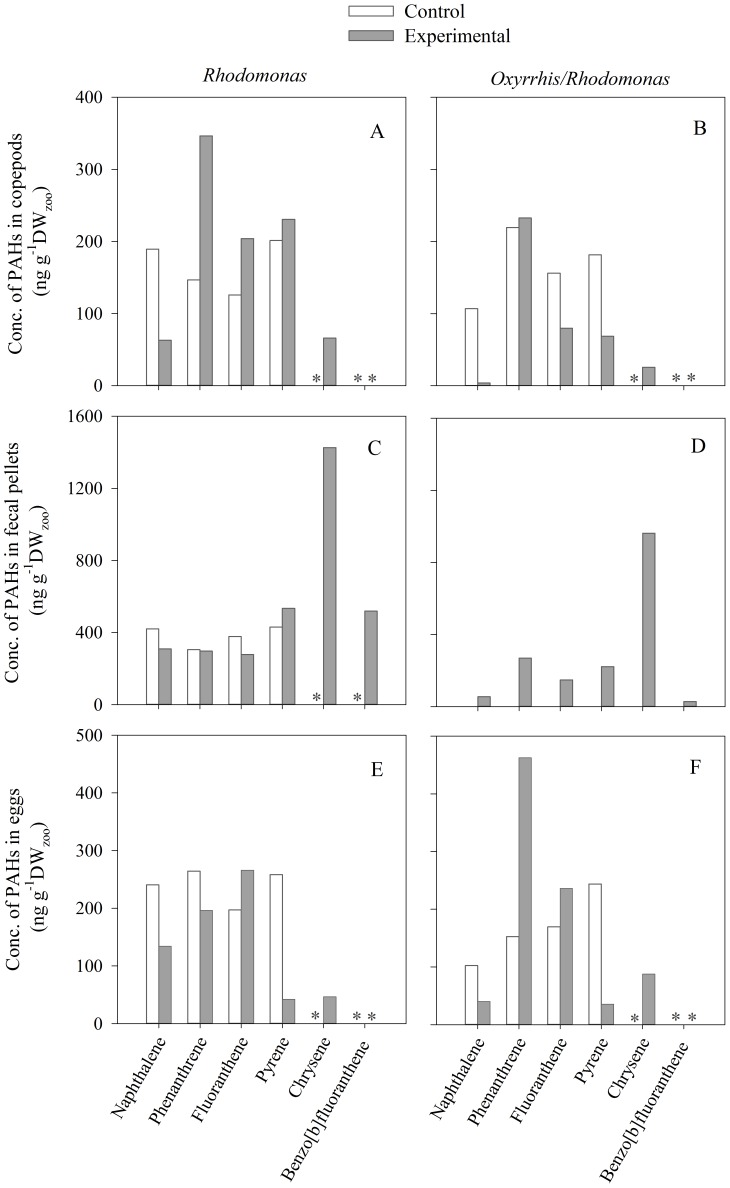
Concentration of PAHs in body tissues (A, B), fecal pellets (C, D) and eggs (E, F) of *Acartia tonsa* feeding on *Rhodomonas* sp.(left column) or *Rhodomonas* sp. plus *Oxyrrhis* marina (right column). Experimental: copepods exposed to oil (5 µl L^−1^). Control: non-exposed copepods. The asterisks indicate the PAH was not detected.

As for natural copepod assemblages, naphthalene, fluoranthene, phenanthrene, pyrene, and chrysene were the main PAHs detected in *A. tonsa* (Fig. 10). However, the concentration of PAHs in the control treatments (non-exposed *A. tonsa*) was relatively higher than those of natural copepod assemblages, except for chrysene and benzo[*b*]fluoranthene that were not detected in both experiments (Fig. 8 and 10). Total concentration of PAHs in *A. tonsa* feeding on *Rhodomonas* was 1.4 times higher in exposed than non-exposed copepods (Fig. 10A). All PAHs showed higher concentrations in experimental treatments than in controls except naphthalene (Fig. 10A). In contrast, PAHs in *A. tonsa* incubated with *Oxyrrhis*/*Rhodomonas* was 1.6 lower in *A. tonsa* exposed to crude oil than in the control treatment (Fig. 10A). The total concentration of PAHs in non-exposed *A. tonsa* incubated with *Oxyrrhis* was similar to those incubated with *Rhodomonas* (664 ng g^−1^ DW). However, the total concentration of PAHs in body tissues of *A. tonsa* incubated with *Oxyrrhis* was >2 times lower than those incubated with *Rhodomomas* (Fig. 10A, 10B). The concentration of all PAHs in body tissues was lower in the experimental treatment with *Oxyrrhis/Rhodomonas* than in that with *Rhodomonas* (Fig. 10A, 10B).

Total concentration of PAHs in fecal pellets of *A. tonsa* incubated with *Rhodomona*s and exposed to crude oil was 2.2 times higher than non-exposed copepods (Fig. 10C). Chrysene and benzo[*b*]fluoranthene were not found in the controls (Fig. 10C). Concentrations of pyrene and, mainly, chrysene and benzo[*b*]fluoranthene were higher in experimental treatments than those of control treatments (Fig. 10C). Unfortunately, data of the PAH concentration in the control treatment with *Oxyrrhis/Rhodomonas* are not available (Fig. 10D). As for *A. tonsa* tissues, the total concentration of PAHs in fecal pellets from *A. tonsa* incubated with *Oxyrrhis/Rhodomonas* was 2 times lower than those incubated with *Rhodomomas* (Fig. 10C, 10D). The concentration of all PAHs in fecal pellets was lower (1.1–18.3 times depending on the PAH) in the experimental treatment with *Oxyrrhis/Rhodomonas* than in that with *Rhodomonas* (Fig. 10C, 10D).

The total concentration of PAHs in eggs of *A. tonsa* incubated with *Rhodomonas* was quite similar in both the control and experimental treatments (Fig. 10E). In contrast, the total concentration of PAHs in eggs of *A. tonsa* incubated with *Oxyrrhis/Rhodomonas* was 1.3 times higher in the experimental treatment than in the control treatment (Fig. 10F). Although the concentration of chrysene and phenanthrene in eggs was 1.9 and 2.4 times, respectively, higher in the experimental treatment with *Oxyrrhis/Rhodomonas* than in that with *Rhodomonas* (Fig. 10E, 10F), there was not a uniform pattern of increasing or decreasing concentration of PAHs in eggs between experimental treatments (Fig. 10E, 10F), contrary to our observations for copepods and fecal pellets (Fig.10A–D).

Bioaccumulation factors (BAFs) in *Acartia tonsa* tissues ranged from 4 to 1023 depending on the type of PAH and the food regime (Table 5). As for natural copepod assemblages, the highest BAF in the tissues of *A. tonsa* was for fluoranthene and pyrene (Table 5). The highest BAF (>5000) was observed in *A. tonsa* fecal pellets for benzo[*b*]fluoranthene (Table 5). BAF of PAH in eggs did not show any clear relation to the food regime (Table 5). BAF for all PAHs in *A. tonsa* tissues and fecal pellets were lower in those incubated with *Oxyrrhis/Rhodomonas* than those incubated with *Rhodomonas* (Table 5).

**Table 5:** Bioaccumulation factor of PAHs in body tissues, fecal pellets and eggs of the copepod *Acartia tonsa* exposed to crude oil (5 µl L^−1^, 48 h, artificial light) with two different food regimes:

*Type of food*	*A.tonsa sample*	Phe	Flua	Pyr	Chr	BbF
*Rhodomonas* sp.	body tissues	66	1023	190	68	*n.d.*
	fecal pellets	–	–	670	1471	5276
	eggs	–	902	–	48	*n.d.*
*Oxyrrhis marina* *Rhodomonas* sp.	body tissues	4	–	–	27	*n.d.*
	fecal pellets	–	–	–	992	288
	eggs	102	874	–	90	*n.d.*

(1) *Rhodomonas* sp. and (2) *Rhodomonas* sp plus *Oxyrrhis marina*. Phenanthrene (Phe), fluoranthene (Flua), pyrene (Pyr), chrysene (Chr), benzo[b]fluoranthene (BbF). Dash indicates no bioaccumulation (concentration in experimental treatment was similar or lower than in respective control treatment). *n.d.* = no detected.

## Discussion

### Oil and Dispersant Exposure Levels

The concentration of crude oil in marine environments after oil spills is highly variable, ranging from a few ppb to hundreds of ppm, depending on many different factors, such as temporal and spatial scales, marine topography and hydrodynamics, and the magnitude of the spill accident. The concentrations of crude oil used in these exposure experiments (5–100 µl L^−1^) are equivalent to 4.2 to 84.5 parts per million (ppm). After oil spills, crude oil in the upper few meters of the water column may reach concentrations of 20–40 ppm or higher [64]. The reported crude oil concentrations following the Deepwater Horizon Oil spill ranged from 0.25 parts per billion (ppb) to 0.22 ppm in coastal and estuaries areas [65], between 1–2 ppm in oil plumes at 1 km depth [66] and from 3.1 to 4500 ppm on Florida beaches [67]. Similarly, reported concentration of total polycyclic aromatic hydrocarbons (PAHs) in water samples during the Deepwater Horizon Oil spill ranged from over 100 µg L^−1^ (ppb) near the wellhead to below detection limit in distant waters [68]. Although total PAHs can reach extreme concentrations in seawater, up to 600 µg L^−1^ [69], [70] and 10,980 µg L^−1^ [71], total PAHs concentration may frequently range from 1 to 150 µg L^−1^ during oil spills [72]–[75]. Considering the total concentration of PAHs in the crude oil was 2.1 µg µL^−1^, the concentration of total PAHs used in our experiments would range from approx. 10.2 to 201 µg L^−1^ (ppb). Shore-based and lab experiments were conducted with an oil concentration of 5 µl L^−1^, corresponding to a total PAH concentration of 10.2 µg L^−1^ (10 ppb), which in the range of concentration commonly found in the water column during oil spills [72]–[75]. Although some crude oil concentrations used in our experiments were in the upper range of observed exposure levels in the field, our studies reflect reasonable/realistic exposure concentrations for mesozooplankton after oil spills, particularly in marine areas close to the oil spill source, upper meters of the water column and coastal waters.

Unfortunately, field measurements of dispersant concentrations in oil spills are scarce, although concentrations up to 13 ppm have been measured in upper surface waters [76]. Also, it generally has been thought that oil dispersant concentrations range from 10 ppm to less than 1 ppm after application [77], [78]. Therefore, the concentration of dispersant used in our experiments (0.25 µl L^−1^, 0.25 ppm) would be a realistic concentration during the clean-up response to oil spills with dispersants.

### Lethal and Sublethal Effects of Crude Oil in Zooplankton

Our results support previous studies that found zooplankton are especially vulnerable to acute crude oil pollution, showing increased mortality and sublethal alterations of physiological activities, e.g, egg production [79]–[82]. Direct comparisons among crude oil toxicological studies are difficult due to the variable composition of crude oils and differences in the methodology and experimental conditions (exposure time, temperature, light regime, etc.). Most published studies have been conducted using the crude oil water soluble fraction (WSF), or certain mixed or individual PAHs. However, oil droplet ingestion may be an important entry of oil in zooplankton [22]–[26], [39]–[41], [83]. The exposure to crude oil may promote zooplankton uptake of PAHs as compared with experiments using WSF or single PAHs [42]. In our experiments, PAHs would have been present in both dissolved and particulate (oil droplet) forms. Toxic effects of naphthalene, the most abundant PAH in crude oil, in zooplankton are frequently observed at much higher concentrations compared to crude oil or the WSF exposure experiments [29]–[30], [33], [36]. This indicates that other PAHs contained in crude oil, (e.g. fluoranthene, pyrene) are more toxic than naphthalene to copepods [29], [36], [84]. It is also important to note that weathered oil generally is less toxic than fresh crude oil because of the loss of volatile fractions [81]. In an open system and under marine hydrodynamics, some of the toxic compounds of the crude oil, such as benzene, toluene, ethyl benzene and xylenes (BTEX) and some PAHs, like naphthalene and acenaphthylene, may be lost by evaporation, reducing the potential toxicity of oil after several days. Considering the total concentration of PAHs in the crude oil was 2.1 µg µL^−1^, the median lethal concentration (31.6 µl of crude oil L^−1^) observed for mesozooplankton communities after short-term oil exposure corresponded to a total PAH concentration of 63.5 µg L^−1^ This concentration is in the lower range of LC_50_ values commonly reported for copepods exposed to WSF in lab studies after 24 h (from ca. 10 µg L^−1^ to >1000 µg L^−1^) [36]–[38], [85]. Although we did not aim to test the effects of oil on single species, we also observed that small copepod species (e.g. *Oithona, Paracalanus*) and copepodites tend to be more sensitive to oil exposure than larger copepods and crustacean larvae, which agrees with other laboratory studies conducted with copepods [38]. Among marine animals, crustaceans are especially sensitive to crude oil exposure [86]–[87]. In general, according to our results and previous research, marine planktonic copepods seem to be more affected by oil pollution than benthic harpacticoid copepods [31], [88]–[90] and other crustaceans [91]–[95]. Therefore, planktonic copepods may be used as a target/indicator group for evaluating and monitoring the environmental impact of oil pollution in marine environments.

Narcosis was one of the sublethal effects that we observed in copepods exposed to crude oil, in agreement with other studies [30], [36], [96]. Narcotic effects in copepods may be associated to both the volatile components of petroleum (BTEX) and the PAHs [29], [36] Although narcosis in copepods is reversible after exposure to unpolluted water [36], if it is prolonged, it may reduce feeding and consequently cause death, or may increase the risk of mortality by predation in nature. Alterations in reproduction, feeding and egestion rates have been commonly observed in copepods exposed to specific PAHs [30], [97]–[98]. However, there is a big discrepancy among studies regarding what physiological rates are affected, and the results vary widely depending on the species and oil exposure concentration. Effects of oil on copepod reproduction depend on both the composition and concentration of petroleum hydrocarbons [99]–[100]. Although in some studies harmful effects to the reproduction of some copepod species has only been found at very high PAH concentrations [33], [99], deleterious effects on reproduction success has also been observed in copepods exposed to low concentration of PAHs, including reduced egg production [36], [85], [101] and reduce/delayed hatching [102]–[103]. Similarly, effects of oil exposure on fecal pellet production rates depend on the species and exposure levels. Likewise, both reduced [33], [85] and unaffected [103] egestion rates have been observed in copepods. Although increased feeding efficiency has been reported in *Calanus finmarchicus* at higher concentrations of naphthalene and WSF oil [104], most studies observed reduced feeding in copepods exposed to high, but sublethal concentrations (>100 µg L^−1^) of WST or naphthalene [30], [33], [36], [97]. However, at lower oil exposure concentrations (<100 µg L^−1^), both reduced [101] and unaffected feeding have been observed in copepods [97], [104]. Reduced ingestion and egestion rates have been related to narcosis or sluggish effects disturbing feeding [30]. In our study, we did not find narcosis effects in *Acartia tonsa* with our experimental conditions (5 µL L^−1^, equivalent to total PAH = 10.2 µg L^−1^, dim light), then reduced fecal pellet production rates or feeding due to narcosis would not be expected. A recent study conducted with *A.tonsa* exposed to low concentrations of oil WSF (15.5 µg L^–1^) showed a significant reduction in egg production rates and a delay in eggs hatching time [85] in agreement with our results (Fig. 7). However, in contrast to this published study [85], we did find a significant effect of oil exposure in *A. tonsa* fecal pellet production rates. The decrease in *A. tonsa* egg production observed in our study was not associated to lower ingestion rates, as reflected in the fecal pellets production rates (no significant differences between treatments, Fig. 7). Reduction of egg production not being associated with reducing feeding rates has been reported for other copepod species exposed to oil [88]. Delayed development associated to oil exposure has also been observed in other crustaceans [105]–[107]. Our results suggest that sublethal oil concentrations may affect the energetics and/or the biochemical processes associated with egg production and embryonic development in copepods. Alterations in the lipid metabolism, including steroid metabolism, may account for energetic and reproduction/developmental anomalies observed in marine crustaceans exposed to petroleum hydrocarbons [107]–[108].

### Effect of Dispersant and Dispersant Treated Oil

Laboratory studies have found that Corexit dispersants are toxic to marine benthic invertebrates and fishes, particularly eggs and early developmental stages [49], [109]–[110]. The limited previous studies on the effects of Corexit dispersant on marine planktonic copepods showed a LC_50_ of 8–12 ppm for *Pseudocalanus minitus* [111] after 48 h exposure to Corexit 9527, and a LC_50_ of 5.2 ppm for *Eurytemora affinis* after 96 h exposure to Corexit 9500A [112]. Chemical toxicity of dispersant is associated with their chemical components, solvents and surfactants. Surfactants can affect the cellular membranes, increasing membrane permeability and causing membrane lysis in marine organisms [113]–[114]. Corexit 9500A was the main dispersant type used to clean up the Deepwater Horizon oil spill in the Gulf of Mexico [115]. Although it is assumed that Corexit 9500A is less toxic than previous dispersant types, recent reports found that Corexit 9500A has similar toxicity to other oil dispersants when mixed with South Louisiana sweet crude oil [116]. Furthermore, Corexit 9500A and oil treated with this dispersant are highly toxic to small planktonic organisms, including mollusk embryos [49], fish eggs and larvae [57], coral larvae [117], and rotifers [118]. We found that Corexit 9500A produce nearly 50% mortality in natural mesozooplankton communities at concentrations of 0.25 ppm (Fig. 5), which is more than one order of magnitude lower than lethal concentrations commonly observed in other marine animals exposed to dispersant [109]–[110], [117]–[118]. This indicates that mesozoplankton communities are highly sensitive to oil dispersant Corexit 9500A.

Several studies have observed the combination of oil and dispersant increased toxicity to marine organisms [57], [117]–[118]. However, studies of the effects of dispersant treated oil on zooplankton communities or copepods are very scarce and sometimes controversial. Linden et al. [119] did not find significant differences in mesozooplankton abundance when exposed to North Sea crude oil and oil treated with Corexit 9550 dispersant. In contrast, Jung et al. [120] observed that zooplankton communities were less affected with crude oil alone than with both crude oil and dispersant, in agreement with our results (Fig. 5). Increased toxicity of dispersant treated oil may be due to additive and/or synergistic effects of oil and dispersant. The dispersant Corexit 9500A may increase the concentration of toxic petroleum hydrocarbons (e.g. PAH) in the water, and consequently, enhance the oil toxicity [121]–[122]. However, in our experiments we found that the toxicity in the dispersant treated oil (72%) would be caused mainly by additive toxicity of oil (mortality = 21%) and dispersant (mortality = 48%) (Fig. 5).

Given the importance of mesozooplankton in marine food webs and their high sensitivity to dispersant and dispersant treated oil, we highly recommend the use of representative planktonic copepods as a target species to evaluate the impact of oil spill chemical cleanup operations in marine environments.

### Bioaccumulation of Polycyclic Aromatic Hydrocarbons in Mesozooplankton

We found that zooplankton can accumulate PAHs when exposed to oil, in agreement with previous studies [20], [39], [41], [43], [123]–[124]. Since we used crude oil emulsions instead of WSF, it is possible that oil droplets could attach to the zooplankton, which has been observed in laboratory and field studies [20]. However, the use of filtration and high pressure washing would substantially remove any attached oil droplets, even though we cannot completely disregard the possibility of attachment of very small oil droplets to zooplankton. The differences in PAH composition between crude oil and contaminated zooplankton (Fig. 2 and Fig. 8), and the PAH concentrations among exposure levels (Fig. 8), support the conclusion that processes other than oil droplet attachment controlled the bioaccumulation observed in our studies. Nevertheless, it is important to note that, in nature, the adhesion of crude oil droplets to zooplankton may be another route of transfer of PAHs up through marine food webs.

The bioaccumulation factors of PAHs reported for zooplankton in oil exposure tests vary widely depending on the species and experimental approach [20], [39], [41], [43], [123]–[124]. Bioaccumulation of a specific pollutant depends on its chemical properties, its bioavailability and the physiology of the organism [125]–[126]. PAHs are lipophilic and their hydrophobicity increases as their molecular weights increase [127]. Because of their lipophilic nature, PAHs are usually accumulated in the lipids of organisms. This would partly explain the differences in PAH concentration observed in zooplankton from our experiments (Tables 4 and 5) compared with those of Arctic copepods with high lipid contents (BAF>5000) [43].

In our experiments, the PAH bioaccumulation factors (BAF) tend to decrease with increasing oil concentration, indicating that bioaccumulation depends on the exposure levels (Table 4). A decrease in BAF with increasing oil concentration may be related to an increase in mortality due to toxic effects of petroleum hydrocarbons, reducing the bioaccumulation, as we observed in our experiments for some PAH (Fig. 9 A–C). However, an inverse relationship between BAF and pollutant exposure level may also relate to processes or mechanisms, other than passive diffusion, that show saturation kinetics [128]. When uptake and removal of petroleum hydrocarbons is due to passive partitioning alone, BAF of PAHs are associated to their lipophilic properties, i.e., octanol–water partition coefficient, K_ow_, with log BAF increasing linearly as increasing log K_ow_ [125], [129]. This pattern has been commonly observed in acute tests conducted with zooplankton exposed to some specific dissolved PAH or WSF [29], [41]. We also found BAF tended to be lower for PAH with low K_ow_ (i.e., naphthalene and phenanthrene), than for PAH with higher K_ow_ (i.e. fluoranthrene, pyrene, chrysene, benzo[*b*]fluoranthrene) (Table 4). Since we used crude oil instead of dissolved petroleum hydrocarbons, the deviations from the linear relationship between log BCF and log K_ow_ observed in our studies may be due to the lower availability of more hydrophobic compounds in the water and the ingestion of oil droplets or prey-oil droplet aggregations. It is important to note that BAF would be also inversely related to the capacity of the organisms to depurate (by excretion or egestion) petroleum hydrocarbons [41], [83], [130]–[131]. Some copepod species are able to metabolize and rapidly biotransform PAHs [132]. The metabolism and depuration rates of a specific PAH depend partially on its chemical properties, e.g. molecular-weight [132]. Then, some petroleum hydrocarbons, such as naphthalene, may be excreted rapidly [41], [129], whereas other PAHs, such as fluoranthene and pyrene, may remain in zooplankton bodies for extended periods [25], [39]–[40], [132]–[133]. PAHs in zooplankton may be also reduced or eliminated by egg production [41]. Oil droplets or some petroleum hydrocarbons have been found into zooplankton fecal pellets in field and laboratory studies [20], [26], [134]–[135]. In the laboratory experiments, we found chrysene and benzo[*b*]fluoranthene, showed low BAF in *Acartia tonsa* despite their high octanol–water partition coefficient, K_ow_ (Table 5). In contrast, we found very high concentrations of these compounds in the fecal pellets (Fig. 10), suggesting chrysene and benzo[b]fluoranthene may be removed from the body via egestion. Field studies found that benzofluoranthenes are frequently accumulated in the marine bottom sediments [136] and Benzo[*b*]fluoranthene was the most abundant PAH in samples of sediments containing mainly copepods feacal pellets [134]. Given their importance in the marine biological fluxes [137]–[139], zooplankton fecal pellets may play a relevant role in the distribution of petroleum hydrocarbons in the sea.

Copepod eggs are rich in lipids, and therefore may potentially accumulate high concentrations of lipophilic contaminants [140]. Although information on the bioaccumulation of PAHs in zooplankton egg is scarce, accumulation of some specific PAH, i.e. fluoranthrene, has been found in copepods eggs [40]. We found bioaccumulation of some petroleum hydrocarbons, such as phenanthrene, fluoranthene, and chrysene in eggs of *Acartia tonsa* exposed to crude oil (Table 5). However, these results should be considered cautiously due to the high concentration of PAH in the control treatments, except for chrysene (Fig. 10). If PAHs are transferred to the next generation through the eggs (e.g. resting eggs), the persistence of PAH in the planktonic communities would be longer than expected for contaminated copepods with short generation times. More investigation is required to evaluate the importance of oil contaminated copepod eggs in the flux and resilience of PAHs in marine systems.

### Influence of Experimental Conditions (UV Exposure, Food) in Crude Oil Toxicological and Bioaccumulation Studies

Oil toxicity in marine organisms may vary widely depending on environmental variables, including temperature [38], salinity [141], light [54]–[55], and turbulence [142]. Among the different extrinsic variables affecting oil toxicity, the influence of UV radiation and food on the toxic effects of oil to zooplankton will be discussed in light of our results.

Ultraviolet radiation (UVR) may increase the toxicity of petroleum hydrocarbons (e.g. PAHs) by 2- to 50,000-fold, depending on the aquatic organism and the type of crude oil or petroleum hydrocarbon [55], [57], [59], [124]. PAHs absorb visible and UV radiation, and therefore are particularly susceptible to photoenhanced toxicity [54]. Photoenhanced toxicity of crude oil may be caused by photosensitization (i.e. bioaccumulated petroleum hydrocarbons act as photoreceptors and transfer light energy to other surrounding biological molecules causing cell and tissue damage) and photomodification (i.e. petroleum hydrocarbons are photochemically transformed into more toxic compounds, such as reactive oxygen species or free radicals, capable of damaging cells) [143]–[144]. Recent studies found that transparent marine organisms, such as fish larvae and embryos [57], [145] and planktonic copepods [59], [124], are particularly sensitive to the combined effects of oil and UVR exposure. We found a moderate increase in toxicity (35%, Fig. 6) compared to other studies [55], [57], [59], [124]. Unfortunately, we were not able to directly measure the UVR during the incubations due to logistic problems. However, our results indicate that, under natural radiation values, UVB increase the toxicity of dispersed crude oil to mesozooplankton communities, which emphasizes the relevance of considering the photoenhanced toxicity in the evaluation of the potential impact of oil spills. For example, translucent/transparent zooplankton, particularly those adapted to live in the upper layers of the water column (neuston) and in intertidal and shallow coastal areas with elevated UVR would be more sensitive to oil pollution.

Many acute toxicological and bioaccumulation studies with zooplankton have been conducted without food [29], [43], [146] following standard protocols (ISO 1999). Nevertheless, zooplankton may take up toxic petroleum hydrocarbons directly, through passive uptake (cutaneous absorption), and/or indirectly, through the ingestion of phytoplankton [41], [83], [131]. The dietary intake of petroleum hydrocarbons is relevant because phytoplankton may accumulate higher concentrations of PAH than zooplankton [41] and BAF of some petroleum hydrocarbons ingested through the diet may be higher than from the dissolved state in seawater [83]. Moreover, some studies have found marine ciliates and pelagic tunicates only ingest oil droplets in presence of phytoplankton [21], [26]. Therefore, starvation conditions would represent unrealistic conditions that may bias the food web mediated interactions between oil and zooplankton.

It is important to note the type of prey used in the tests may play an important role in the toxicity of crude oil to zooplankton. Under natural conditions, planktonic communities are composed of many organisms including phytoplankton, protozoan and metazoans. Both phytoplankton and protozoans are part of the metazoans diet (e.g. copepods).The protozoan *Oxyrrhis marina* is a high quality prey for copepods in term of essential lipids and they may enhance the copepod growth and reproduction by trophic upgrading [148]; this would explain the increase in egg production of *Acartia tonsa* with *Oxyrrhis marina* observed in our experiments (Fig. 7). We observed differences in sublethal effects (reduced egg production, delayed hatching) (Fig. 7) and bioaccumulation of PAHs depending on the absence or presence of protozoan *Oxyrrhis marina* in the water (Fig. 10). *O. marina* could remove oil from the water column by both passive uptake of dissolved petroleum hydrocarbons and by ingestion of oil droplets and *Rhodomonas* contaminated with PAHs. This would reduce the oil availability for *Acartia tonsa*, reducing their potential toxicity and bioaccumulation of PAHs, as observed in our study. Unfortunately, there are no available data on the uptake and bioaccumulation of petroleum hydrocarbons by heterotrophic dinoflagellates. Although the abundance of *Oxyrrhis marina* in nature is lower than in our experiments, natural concentrations of heterotrophic flagellates together are commonly higher than in our experiments [149]–[150]. Note that heterotrophic flagellates may have a higher tolerance to oil pollution than mesozooplankton [151], and the standing stock of protozoan consumed by metazooplankton is very usually very low in nature (<1%) [150], [152]. Therefore, this suggests protozoans may play an important role in the toxicity and fate of petroleum in the sea.

Overall, our results emphasize the importance of experimental conditions in the crude oil toxicity tests. More experiments (e.g. mesocosms) mimicking the natural environment (e.g. natural microbial assemblages, sunlight, turbulence, etc.) are required to better understand the effects of oil spills on zooplankton communities and the transfer of petroleum hydrocarbon in marine food webs.

### Ecological Implications of the Interactions between Crude Oil and Zooplankton

Impact of oil spills on planktonic communities depends on many physical, chemical and biological factors, and therefore the effects of oil pollution on zooplankton would vary depending on the circumstances of each spill accident [153]. Overall, given the pivotal role of zooplankton in marine environments, harmful effects of oil in zooplankton communities would strongly affect fish production and benthic invertebrate recruitment. In agreement with other acute toxicological studies, oil pollution has negative short-term impacts on zooplankton, resulting in a significant decrease in zooplankton abundance and biomass, and changes in zooplankton composition after oil spills [154]–[156]. It has been suggested that copepods may reduce their exposure to oil due to their ability to avoid oily patches [157]. However, even if copepods are able to detect petroleum hydrocarbons [95], [158], their capacity to avoid crude oil may be limited due to marine hydrodynamics, which may force zooplankton communities into highly polluted waters masses or coastal areas. The frequent observation of ingested oil droplets in zooplankton collected from the field after oil spills suggests low capability by zooplankton to avoid oil patches under natural hydrodynamic conditions.

During the DWH oil spill, more than 1.7 million gallons of chemical dispersants, mainly Corexit 9500A, were applied at the sea surface and on the seafloor near the wellhead [159]. The use of dispersant in oil spills enhances the formation of small oil droplets, promoting bacterial biodegradation, but at the same time, also increases the potential exposure of oil to pelagic organisms. The application of dispersants may increase the negative impact of oil spills to planktonic communities due to its high toxicity to mesozooplankton as observed in this study. Corexit 9500A is also toxic, more toxic than oil alone, for tintinnid ciliates and dinoflagellates (Almeda et al., unpublished data). Hence, less toxic dispersants are required to reduce their impact on planktonic organisms. Moreover, although it is thought that dispersants are rapidly diluted and degraded in marine environments [49], a recent study [160] found a slow degradation of Corexit 9500A dispersant ingredients in deep waters after the DWH spill. These results accentuate the importance of further studies with key planktonic organisms (e.g., copepods, microzooplankton) from surface and deep waters for a better understanding of the impact of dispersants on planktonic communities and, consequently, a better evaluation of the pros and cons of the application of dispersants in the sea after an oil spill.

Given the capacity of zooplankton to accumulate toxic petroleum hydrocarbons in tissues, fecal pellets and eggs, planktonic communities may play an important role in distribution of toxic petroleum hydrocarbons in marine ecosystems after oil spills [41], [131], [161]. Since zooplankton are the main food of many marine animals, PAHs may move to higher trophic levels, including pelagic and benthic communities [9]. Sedimentation of fecal pellets produced in the photic zone represents one of the main mechanisms of the vertical flux of particulate organic matter in the ocean [137]. Likewise, fecal pellets may represent part of the diet of coprophagous copepods in the epipelagic zone and an important food source to the deep-sea and the benthos [138]–[139]. Therefore, zooplankton fecal pellets may also be an important vector in the biological flux of petroleum hydrocarbons in the water column and toward the benthic food web. The accumulation of PAHs in copepods eggs (e.g. resting eggs) would increase resilience of PAH in marine systems. Overall, knowledge on transfer and bioaccumulation of PAH in marine food webs mediated by zooplankton is required to evaluate the fate of oil pollution and their impact in marine environments.

Although negative short term effects of oil pollution to zooplankton are generally accepted, the long term effects of oil pollution and the capacity of recuperation of zooplankton communities are still important questions of debate. Some studies found that zooplankton communities seem to reestablish after several weeks/months after an oil spill, indicating a high capacity for recovery [161 163]. However, marine hydrodynamics and the high natural variability and patchiness in zooplankton abundance may mask the real impacts of oil on zooplankton communities [164]. In open waters, new planktonic communities from unaffected oil areas may be transported to the affected area by the mixing of water masses. However, the recovery of zooplankton communities might not be equally efficient in all ecosystems as it would depend upon the affected area and the planktonic community composition. Zooplankton communities from coasts, estuaries, and enclosed bays with restricted hydrodynamics, would be more susceptible to long term effects than zooplankton communities living in open water with high hydrodynamics, where mixing and dilution may reduce the time and exposure levels. Some reports also suggest that zooplankton may be minimally affected by oil spill pollution over the long term [153], [157], [165] due to their short generation times and high fecundity. However, the impact of oil may depend of the life history of the specific zooplankter. For instance, some species of calanoid copepods in mid and high latitudes reproduce mainly during specific seasons, producing resting eggs that remain in the sediments until the following year [166]. Similarly, spawning of marine benthic invertebrates in mid and high latitudes shows strong seasonality, with specific peaks of egg and planktonic larvae production. If an oil spill affected these organisms during their reproduction season, reduced egg production and larval survival may affect the recruitment for the following year, and therefore the population dynamics of planktonic and benthic communities. These are just a few examples that highlight the complexity of evaluating the long-term effects of oil spills on zooplankton communities, and their ecological impact in marine environments.

### Main Conclusions

Our experiments indicate zooplankton are especially vulnerable to acute crude oil exposure, showing increased mortality and sublethal alterations of physiological activities (e.g., reduced egg production and delayed hatching). We also found that the chemical dispersant Corexit 9500A was highly toxic to coastal mesozooplankton communities, more toxic than oil alone. Bioaccumulation of certain polycyclic aromatic hydrocarbons (PAHs) was observed in natural mesozooplankton communities, copepods, eggs and fecal pellets exposed to crude oil, suggesting zooplankton may play in important role of the distribution and turnover of petroleum hydrocarbons in marine environments after oil spills.

We found that both environmental (e.g. sunlight radiation) and biological (e.g. microbial community composition) factors affect the interactions between crude oil and mesozooplankton. Natural UVB radiation exposure increased the toxicity of crude oil on mesozooplankton communities. On the other hand, the presence of protozoans in the water reduced the toxic effects of crude oil and the bioaccumulation of PAHs in copepods. These results highlight that further experiments that mimic the natural environment (e.g., mesocosms) are required to accurately evaluate the toxic effects and bioaccumulation of petroleum hydrocarbons in zooplankton.

Overall, our research emphasizes that more knowledge of oil-zooplankton interactions (e.g., zooplankton ingestion of crude oil, transfer of PAHs in food webs as mediated by zooplankton) with key planktonic organisms (e.g., copepods, meroplankton, microzooplankton) are needed for a better understanding of the impact of oil spills and the fate of petroleum hydrocarbons in marine environments.
